# Supplementary material for: The Systems Biology Research Tool: evolvable open-source software
Source: BMC Syst Biol. 2008 Jun 29;2:55. doi: 10.1186/1752-0509-2-55 (PMC2446383; doi:10.1186/1752-0509-2-55)
Supplement: Additional file 1 — SBRT Archive. An archive of the current version of the Systems Biology Research Tool. [file 1752-0509-2-55-S1.zip › sbrt-1.4.0/doc/developers_guide/api/sbrt/kernel/math/optimization/class-use/ProgramSolverStatus.html]

Uses of Class sbrt.kernel.math.optimization.ProgramSolverStatus


|  |  |  |
| --- | --- | --- |
| **Overview** \| **Package** \| **Class** \| **Use** \| **Tree** \| **Deprecated** \| **Index** \| **Help** | |  |
| PREV   NEXT | **FRAMES**    **NO FRAMES**     **All Classes** |  |


---


## **Uses of Class sbrt.kernel.math.optimization.ProgramSolverStatus**

| Packages that use ProgramSolverStatus | |
| --- | --- |
| **sbrt.ext.cplex** | Provides a class that allows communication with CPLEX. |
| **sbrt.ext.glpk** | Provides a class that allows communication with GLPK. |
| **sbrt.kernel.fba.comp** | Provides classes and interfaces for performing Flux Balance Analysis computations. |
| **sbrt.kernel.math.optimization** | Provides classes and interfaces for creating objects relevant to solving optimization problems. |

Uses of ProgramSolverStatus in sbrt.ext.cplex

| Methods in sbrt.ext.cplex that return ProgramSolverStatus | |
| --- | --- |
| `ProgramSolverStatus` | `CplexSolver.getStatus()`             Returns the status of the most recently attempted optimization. |

Uses of ProgramSolverStatus in sbrt.ext.glpk

| Methods in sbrt.ext.glpk that return ProgramSolverStatus | |
| --- | --- |
| `ProgramSolverStatus` | `GlpkSolver.getStatus()`             Returns the status of the most recently attempted optimization. |

Uses of ProgramSolverStatus in sbrt.kernel.fba.comp

| Methods in sbrt.kernel.fba.comp that return ProgramSolverStatus | |
| --- | --- |
| `ProgramSolverStatus` | `FbaOptException.getStatus()`             Returns the status of the attempted optimization. |

Constructors in sbrt.kernel.fba.comp with parameters of type ProgramSolverStatus

`FbaOptException(MathExpr<?> objectiveFunction,
OptimizationSense sense,
ProgramSolverStatus status)`
  
          Constructs a new FBA optimization exception.

`FbaOptException(MathExpr<?> objectiveFunction,
OptimizationSense sense,
ProgramSolverStatus status,
java.lang.String message)`
  
          Constructs a new FBA optimization exception.

Uses of ProgramSolverStatus in sbrt.kernel.math.optimization

| Methods in sbrt.kernel.math.optimization that return ProgramSolverStatus | |
| --- | --- |
| `ProgramSolverStatus` | `ProgramSolver.getStatus()`             Returns the status of the most recently attempted optimization. |
| `static ProgramSolverStatus` | `ProgramSolverStatus.valueOf(java.lang.String name)`             Returns the enum constant of this type with the specified name. |
| `static ProgramSolverStatus[]` | `ProgramSolverStatus.values()`             Returns an array containing the constants of this enum type, in the order they're declared. |

---


|  |  |  |
| --- | --- | --- |
| **Overview** \| **Package** \| **Class** \| **Use** \| **Tree** \| **Deprecated** \| **Index** \| **Help** | |  |
| PREV   NEXT | **FRAMES**    **NO FRAMES**     **All Classes** |  |


---
